# Anti-asthmatic effect of pitavastatin through aerosol inhalation is associated with CD4+ CD25+ Foxp3+ T cells in an asthma mouse model

**DOI:** 10.1038/s41598-017-06476-6

**Published:** 2017-07-20

**Authors:** Songquan Wu, Ruhui Yang, Guangli Wang

**Affiliations:** College of Medicine and Health, Lishui University, 1 Xueyuan Road, Lishui, 323000 Zhejiang Province P.R. China

## Abstract

Statins are competitive inhibitors of 3-hydroxy-3-methylglutaryl-CoA (HMG-A) reductase, and studies have shown that statins also have anti-inflammatory and immunomodulatory properties. The purpose of this study was to investigate the anti-asthmatic effects of pitavastatin, a type of statin, in an asthma mouse model. Mice were sensitized and challenged with ovalbumin (OVA) to establish the asthma model. These mice were then treated with inhaled pitavastatin (5 mg/kg) or dexamethasone (2 mg/kg), the latter of which served as a positive control. The results of the study showed that pitavastatin reduced allergen-induced increases in airway resistance and alleviated bronchial tube thickness and goblet cell hyperplasia in lung tissues. In addition, the results showed that pitavastatin inhibited OVA-induced increases in eosinophil counts and total inflammatory cell counts in bronchoalveolar lavage fluid (BALF) and increased the percentage of CD4+ CD25+ Foxp3+ Treg in the BALF of asthmatic mice. IL-4 and IL-17 levels were decreased, whereas IFN-γ levels were significantly increased in the BALF of pitavastatin-treated mice compared with the BALF of OVA-challenged mice. These results suggest that pitavastatin has potential as a therapy for allergic airway disease and that its effects are associated with its ability to regulate CD4+ CD25+ Foxp3+ T cell counts.

## Introduction

Asthma is a common chronic inflammatory disease of the airway and is also a leading cause of morbidity among children and adults worldwide^1, 2^. Chronic airway inflammation and airway hyper-responsiveness play important roles in the pathogenesis of asthma^3, 4^, and genetic and immunological analyses of atopic individuals have shown that Th2 lymphocytes play a key role in airway inflammation initiation and maintenance^5^.

Foxp3, a transcription factor, is considered a main regulator of the development and function of CD4+ CD25+ regulatory T cells^6^, which are now recognized as key players in many physiologic and pathophysiologic processes, including autoimmune diseases, allergic responses and airway remodelling^7–9^.

Statins inhibit 3-hydroxy-3-methylglutaryl-coenzyme A (HMG-A) reductase, and some recent experimental studies have shown that statins can reduce inflammatory cell infiltration and decrease the number of eosinophils in bronchoalveolar lavage fluid (BALF) in animal models of asthma, as well as inhibit airway smooth muscle proliferation and contraction *in vitro* ^10, 11^. More recently, clinical trials have shown that short-term treatment with statins increases lung function, enhances the anti-inflammatory effects of corticosteroids in patients with asthma, and improves the results of the Asthma Control Questionnaire and Asthma Quality of Life Questionnaire^12–14^. Moreover, these studies have shown that statins improve outcomes in patients with asthma who are receiving inhaled corticosteroid therapy and exert multiple beneficial effects in such patients through their pleiotropic anti-inflammatory properties^15, 16^.

Pitavastatin is a novel HMG-CoA reductase inhibitor whose cholesterol-lowering effect is stronger than that of other statins currently in use. Previous studies have shown that pitavastatin inhibits vascular smooth muscle cell proliferation in *vitro*, regulates helper T-cell differentiation and ameliorates autoimmune myocarditis in mouse models^17, 18^. However, to date, the effects of pitavastatin on asthma and T lymphocyte differentiation have not been well studied. Long-term administration of high doses of oral steroids may have adverse effects^19^. Thus, delivering hydrophilic statins by the inhaled route would be advantageous for patients, as it would ensure that pulmonary medication concentrations are high and systemic medication concentrations are low and thus enable patients to receive efficient and effective therapy for their diseases while avoiding unwanted therapy-related side effects^20^. Studies have shown that inhaled pravastatin is beneficial for the treatment of asthma^21^. Thus, in this study, we aimed to examine the effects of inhaled pitavastatin in a murine model of allergic asthma. We also assessed the changes in CD4+ CD25+ Foxp3+ cell counts and interleukin (IL-4 and IFN-γ) secretion in BALF elicited by pitavastatin treatment. This is the first study to show that the protective effects of pitavastatin against asthma are associated with CD4+ CD25+ Foxp3+ T cells. The findings of the study may open new avenues for the treatment of asthma-related airway inflammation and the development of improved asthma drug formulations.

## Materials and Methods

### Animals and reagents

Specific pathogen-free BALB/c female mice aged approximately six weeks and weighing 18 g to 22 g were provided by Shanghai Slack Laboratory Animal Co., Ltd. The mice were housed in a temperature-controlled room under a 12 h dark/light cycle and were allowed access to food and water ad libitum. This study was conducted in strict accordance with the recommendations in the Guide for the Care and Use of Laboratory Animals of the National Institutes of Health, and its protocol was approved by the Animal Research Ethics Board of the Lishui University (Lishui, Zhejiang Province, China. Permit Number: 0601–2013). All surgeries were performed under sodium pentobarbital anaesthesia, and all efforts were made to minimize suffering. Pitavastatin sodium was purchased from the National Institutes for Food and Drug Control (China) and was prepared with 1 mg/mL sterile phosphate buffered solution (PBS). The pH of the drug was adjusted to 7.4, and its total volume was corrected to 1 mL. The stock solution was diluted to the appropriate concentration in PBS immediately before use. Chick ovalbumin (OVA) was purchased from Sigma (USA), and aluminium hydroxide gel was purchased from Imject Alum (Thermo Scientific Inc., Germany). Dexamethasone sodium was purchased from Cisen Pharmaceutical Co., Ltd. (Ji Ning, China).

### Mouse models of acute asthma

The mouse model was established using a traditional protocol, as previously described^22, 23^. Briefly, allergic asthmatic reactions and airway remodelling were induced in the abovementioned mice using OVA. Specifically, the mice were initially sensitized through intraperitoneal injections of PBS with 25 μg of OVA in 1 mg of aluminium hydroxide gel in 0.2 mL of PBS, pH 7.4, on days 0, 7, and 14 of the study. The mice were subsequently randomized into groups that were repeatedly administered nebulized 5% OVA in PBS or PBS alone by an ultra-sonic nebulizer with an aerosol chamber (Yuyue Medical Equipment & Supply Co., Ltd., Shanghai, China) from days 15 to 21 of the study. The drug was administered for 30 minutes at a time for 7 consecutive days. The asthmatic mice were then divided into three groups (15 mice per group). The pitavastatin-treated group received 5 mg/kg pitavastatin sodium through an ultra-sonic atomizer at the same time each day. The dexamethasone-treated group received 2 mg/kg dexamethasone, which was used as a positive control, through aerosol inhalation. The asthma group received pitavastatin sodium in 10 mL of PBS, pH 7.4, at the same time each day. The PBS group received PBS, which was used as a negative control, via the same route in which OVA was administered.

### Measurement of airway resistance

On day 21, five mice from each group were anesthetized via intraperitoneal (i.p.) injections of 300 mL of pentobarbital sodium (60 mg/kg) before undergoing tracheostomy tube insertion. Airway resistance and compliance measurements were performed using a FinePointe RC system (Buxco Research Systems, Wilmington, NC). The mice were subsequently challenged with aerosolized PBS (baseline) before being treated with acetylcholine at the following ascending doses: 0, 1, 2, 4, 8, and 16 mg/mL. Average compliance values were recorded during a 3-min period following each challenge.

### Bronchoalveolar lavage fluid (BALF) collection and inflammatory cell counts

On day 21, the mice were anesthetized with i.p. injections of 300 mL of pentobarbital sodium (60 mg/kg), and their thoracic cavities were carefully opened. Their tracheas were exposed, and BALF was collected by cannulation of the right principal bronchus. The BALF was subsequently lavaged first with 1 mL and then with 0.8 mL of PBS. Approximately 85% to 90% of the instilled volume was recovered, after which the lavage samples from each mouse were stored on ice. The cell pellets were then resuspended in PBS and stained with trypan blue, and the numbers of nucleated cells were counted in at least five squares of a haemocytometer. Differential cell counts for eosinophils, macrophages, lymphocytes and neutrophils were performed on smears comprising at least 400 cells, which were prepared with a cytocentrifuge and stained with H&E.

### CD4+ CD25+ Foxp3+ Treg cell analysis in BALF by flow cytometry

For flow cytometric analysis, BALF was centrifuged at 1000 × *g* for 5 min at 4 °C. After centrifugation, the cell pellets were suspended in 100 µL of flow cytometry staining buffer with 0.125 μg of anti-mouse CD4 and 0.06 μg of anti-mouse CD25 antibody before being incubated in the dark for 30 min at 4 °C. The pellets were then rinsed twice with flow cytometry staining buffer before being fixed in 1 mL of permeabilization working solution suspension. The cells were subsequently incubated in the dark overnight before being treated with 0.5 µg of Fc blockers (CD16/32) and then incubated in the dark for 15 min at 4 °C. The cells were subsequently treated with 0.5 µg of anti-mouse Foxp3 antibody (or an antibody equivalent to the control antibody) and incubated for 30 min under the appropriate conditions. For each group of five experimental sample data points, the lymphocyte community in the FSC− A/SSC− A scatterplot was selected, CD4+ T cells were selected through the CD4 lymphocyte community/SSC– H set door, and CD4+ CD25+ Foxp3+ Treg cells were divided into separate communities comprising CD25/CD4+ T cells and Foxp3 isotype controls.

### Detection of IL-4 and IFN-γ mRNA expression in BALF

Reverse-transcription polymerase chain reaction (RT-PCR) was used to determine IL-4 and IFN-γ mRNA expression levels in BALF cells. Total RNA was extracted from BALF cells using Trizol reagent (Invitrogen, USA), according to the manufacturer’s instructions. An IQ SYBR Green SuperMix PCR Array Kit was purchased from Bio-Rad (USA). Two micrograms of extracted RNA was converted to cDNA by MMLV-reverse transcriptase (Fermentas, CAN), which was used according to its manufacturer’s instructions. The cDNA was amplified using the following forward and reverse primers as previously described^24, 25^: IL-4, forward: 5′-GGTCTC A ACCCCCAGCTAGT-3′, and reverse: 5′-GCCGATGATCTCTCTCAAGTGAT-3′; IFN-γ, forward: 5′-CACGGCACAGTCATTGAAAG-3′ and reverse: 5′-ATCAG CAGCGACTCCTTTTC-3′; and β-actin, forward: 5′-GAGACCTTCAACACCCCAGC-3′ and reverse: 5′-ATGTCACGCACGATTTCCC-3′. The mouse β-actin housekeeping gene was used as an internal control. The primers were designed and synthesized at Shanghai Generay Biotech (Shanghai, China). The reaction was performed, and its results were analysed by a CFX Connect Real-Time PCR System (Bio-Rad, USA). The relative expression levels of the mRNA in each sample were calculated by normalizing the threshold cycle (Ct) value to the Ct value of the β-actin housekeeping gene using the 2^−ΔΔCt^ method. These levels were expressed in arbitrary units.

### Measurement of IL-4, IL-17, and IFN-γ protein expression by enzyme-linked immunosorbent assay (ELISA)

BALF was centrifuged at 1000 × *g* for 5 min at 4 °C. After centrifugation, IL-4, IL-17, and IFN-γ protein expression levels in the BALF supernatant were measured using a sandwich ELISA Kit (USCN, Life Science Inc., China), according to the manufacturer’s instructions. Samples were read at 450 nm using a SpectraMax Plus 384 microplate reader (Molecular Devices) and SoftMax Pro software.

### Western blot analysis of IFN-γ and IL-4

On day 21, the total protein from the left lung of each mouse was prepared under reducing conditions using 4% to 12% Bis-Tris SDS-PAGE gels before being blotted and detected using anti-IFN-γ (sc-52557, Santa Cruz) and anti-IL-4 antibodies (0.1 μg/mL, ab11524; Abcam). Protein expression analysis was performed using ImageJ 1.44 Quant software.

### Histology of lungs

For histological evaluation of the mouse lung tissue specimens, we fixed the left lung of each mouse in 10% buffered formalin. The fragments were then dehydrated, cleared, and embedded in paraffin. The whole lung was serially sectioned (3-to-4 μm thick), stained with H&E for pathological analysis, and stained with periodic acid-Schiff (PAS) for goblet cell detection. The degree of peribronchial and perivascular inflammation was evaluated according to a subjective scale ranging 0 to 4^19, 20^. Specifically, the degree of cell infiltration in the above tissues was scored as follows: 0, no cells; 1, a few cells; 2, a ring of cells with a depth of one cell; 3, a ring of cells with a depth of two to four cells; and 4, a ring of cells with a depth of more than four cells. Reticular basement membrane thickness was measured by image analyses of multiple randomly selected tissue sections, each of which comprised 30 analysis points, using an Olympus software microscope system. Repeat measurement error was assessed by performing multiple measurements of a single membrane area in four subjects, as previously described^26^.

The degree of goblet cell hyperplasia in the airway epithelium was quantified according to the following five-point system: 0, no goblet cells; 1, <25% of the cells in the epithelium are hyperplasic; 2, 25–50% of the cells in the epithelium are hyperplastic; 3, 50–75% of the cells in the epithelium are hyperplastic; and 4, >75% of the cells in the epithelium are hyperplastic. Five randomly distributed left lung airway sections were analysed in each mouse, and an average score was calculated by summing the scores for each of the five fields.

### Statistical analysis

The data are reported as the mean ± S.D. Statistical significance was determined by ANOVA followed by Tukey’s correction for multiple comparisons or Student’s two-tailed t-test for independent means. Non-parametric analyses were performed using Kruskal-Wallis one-way analysis. All analyses were performed using SPSS 11.0 for Windows (SPSS) software. *P* values less than 0.05 were considered statistically significant.

## Results

### Effect of pitavastatin inhalation on acetylcholine-induced airway hyper-reactivity (AHR)

**Figure 1:**
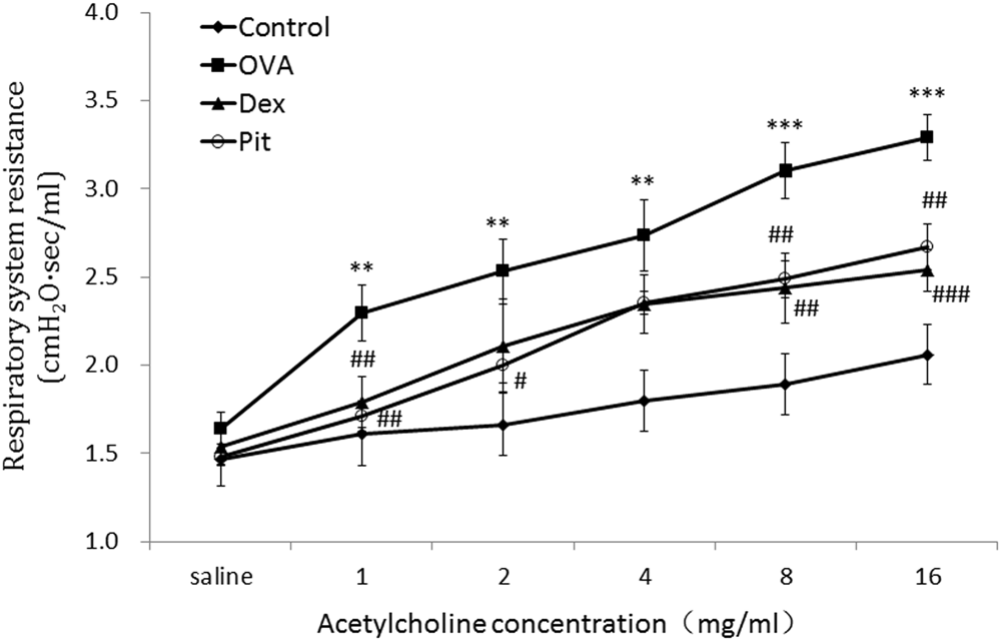
Effect of inhaled pitavastatin therapy on acetylcholine-induced AHR. Airway resistance measurements were performed using a FinePointe RC system. Mice were challenged with aerosolized PBS (baseline) before being treated with acetylcholine at the following ascending doses: 0, 1, 2, 4, 8, and 16 mg/mL. OVA: ovalbumin; Pit: pitavastatin; Dex: dexamethasone. The data are expressed as the mean ± SD (*n* = 6–8 per group). **P < 0.01 compared with the control group; ***P < 0.001 compared with the control group; ^#^ *P* < 0.01 compared with the OVA group; ^##^ *P* < 0.01 compared with the OVA group.

Airway function was assessed by measuring the changes in lung resistance and compliance elicited by acetylcholine inhalation, which induced bronchoconstriction. The measurements were performed on individual OVA primed/challenged mice treated with and without pitavastatin. The airway resistance and compliance measurements for each of the four groups analysed herein are shown in Fig. 1. Airway resistance was increased in OVA primed/challenged mice compared with control mice. Remarkably, pitavastatin-treated mice displayed decreases in airway resistance that were almost equivalent to those displayed by dexamethasone-treated mice. These results indicated that pitavastatin reduces several AHR parameters.

### Effect of pitavastatin on the total and differential cell numbers in BALF

**Figure 2:**
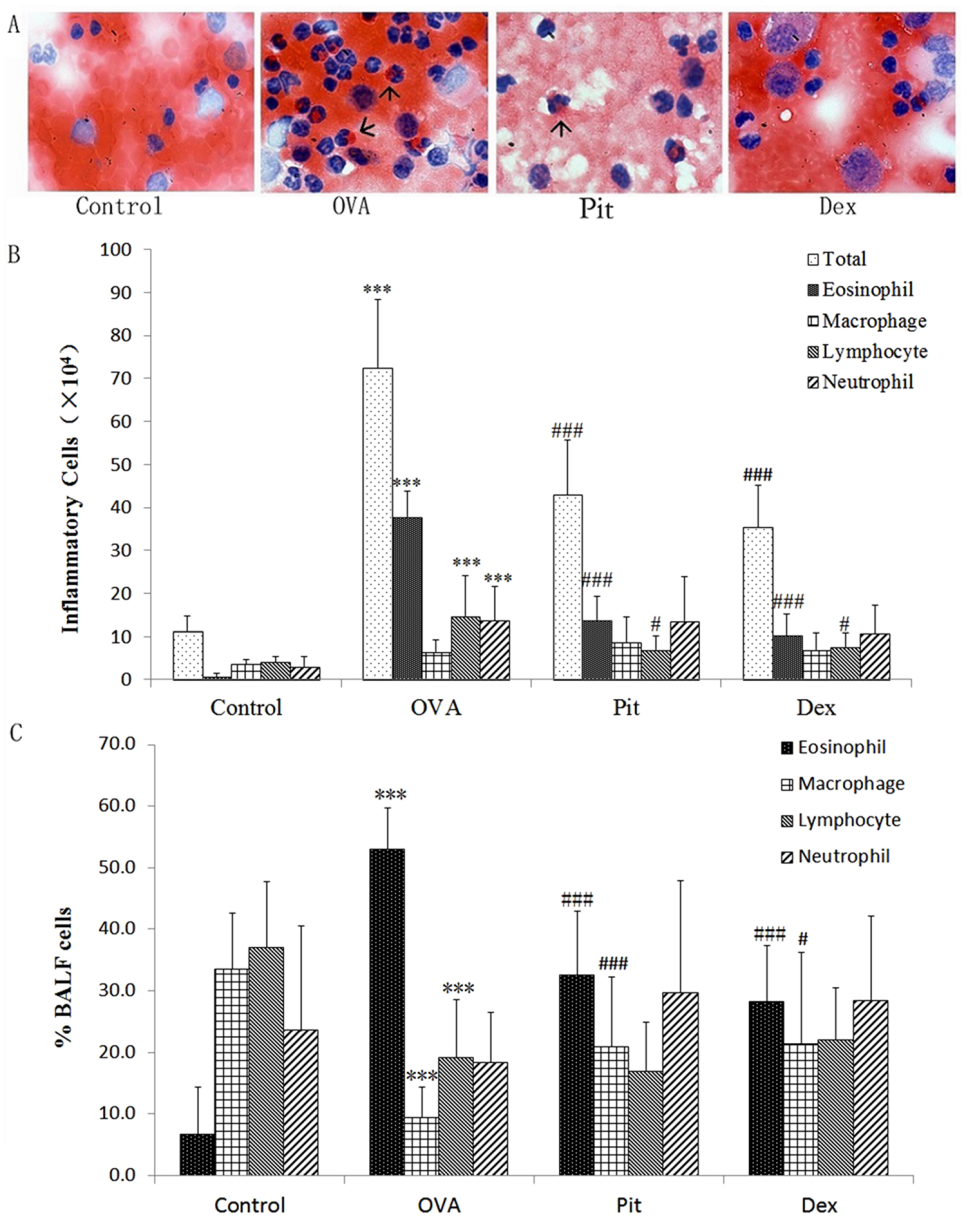
Effect of pitavastatin inhalation on the total and differential cell counts in BALF. The total number of inflammation-related BALF cells was counted using a haemocytometer, and differential cell counts in BALF were determined by staining tissue slides with H&E. Control: Lung sections from mice treated with aerosolized PBS. OVA: Lung sections from mice challenged with OVA and treated with PBS. Pit and Dex: Lung sections from mice challenged with OVA and treated with pitavastatin and dexamethasone, respectively. Values are expressed as the mean ± SD (*n* = 10 per group). ***P < 0.001 compared with the control group; ^#^ *P* < 0.01 compared with the OVA group; ^###^ *P* < 0.001 compared with the OVA group. (**A**) Representative images (400×) of H&E-stained BALF samples. (**B**) Changes in the number of inflammatory cells in the BALF of each group of mice with asthma. (**C**) Percentages of eosinophils, macrophages, lymphocytes, and neutrophils.

Analysis of the inflammatory cells in the BALF samples revealed that total cell numbers were significantly increased by OVA sensitization. However, total cell numbers were reduced by treatment with 5 mg/kg pitavastatin. Specifically, the eosinophil percentage was higher in the OVA-challenged group than in the control group and lower in the pitavastatin-treated group than in the OVA-challenged group. Administration of 2 mg/kg dexamethasone also significantly reduced the total and differential cell counts in BALF in the corresponding group compared with the OVA-challenged group (Fig. 2).

### Pitavastatin treatment increases the numbers of CD4+ CD25+ Foxp3+ cells of BALF

**Figure 3:**
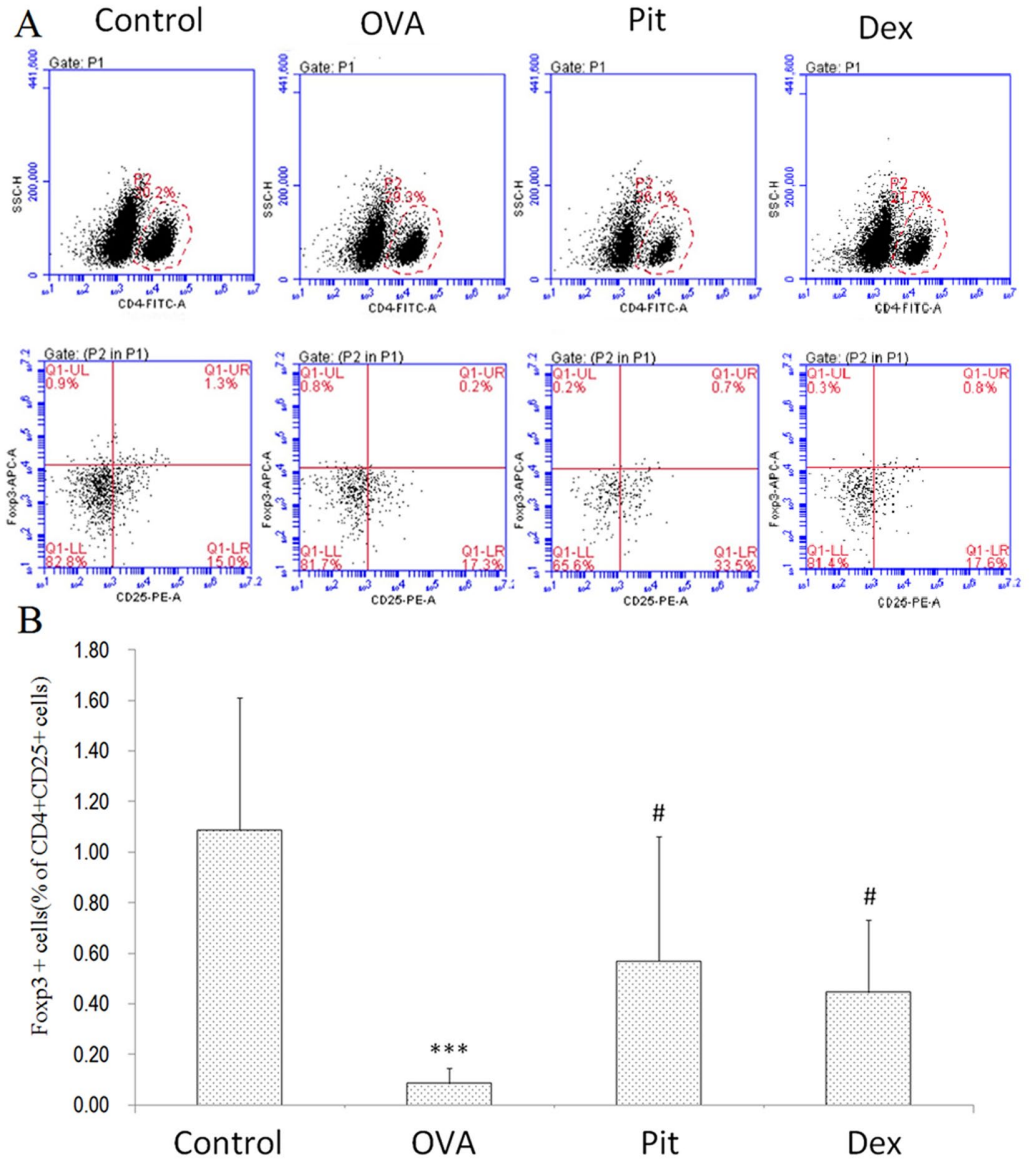
Pitavastatin treatment increases the numbers of CD4+ CD25+ Foxp3+ cells in BALF. Cell suspensions were isolated from the BALF of mice in the control, OVA-challenged, pitavastatin-treated, and dexamethasone-treated groups. The cells were stained with labelled antibodies specific for CD4, CD25, and FoxP3. (**A**) Representative flow cytometry plots representing the proportions of CD4+ CD25+ and CD25+ FoxP3+ T cells (examined on gated CD4+ cells). (**B**) Relative proportion of CD4+ CD25+ Foxp3+ Treg cells. The results represent the mean ± SD (n = 6–8). Control: Lung sections from mice treated with aerosolized PBS. OVA: Lung sections from mice challenged with OVA and treated with PBS. Pit and Dex: Lung sections from mice challenged with OVA and treated with pitavastatin and dexamethasone, respectively. **P < 0.01 compared with the control group; ^#^ *P* < 0.05 compared with the OVA group.

Cell suspensions were isolated from the BALF samples of mice from the control, OVA-challenged, pitavastatin-treated, and dexamethasone-treated groups and stained with labelled antibodies specific for CD4, CD25, and FoxP3. The mice in the OVA-challenged group displayed significantly decreased numbers of CD4+ CD25+ Foxp3+ cells in their BALF compared with the mice in the control group. However, the mice in the pitavastatin-treated group displayed a significantly increased percentage of CD4+ CD25+ Foxp3+ cells in their BALF compared with the mice in the OVA-challenged group (pitavastatin-treated: 0.74 ± 0.42%; OVA: 0.14 ± 0.06%; *P* < 0.05) (Fig. 3).

### Lung histology and quantitative image analysis

**Figure 4:**
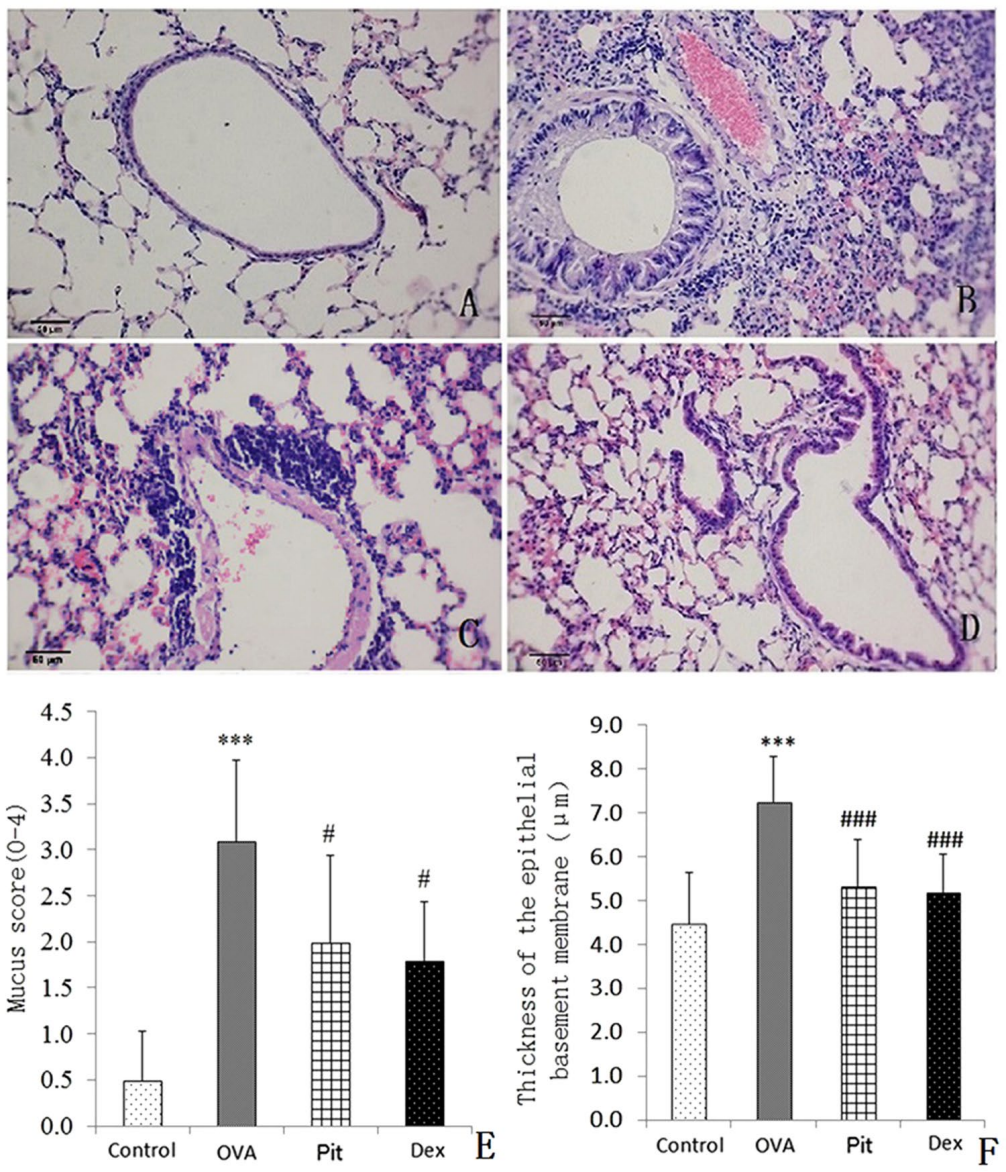
Effects of pitavastatin on OVA-induced lung histological changes, as determined by H&E staining (100×). (**A**) Control mice treated with aerosolized PBS. (**B**) Mice challenged with OVA and treated with PBS showing a peribronchial inflammatory infiltrate consisting mainly of eosinophils and lymphocytes. The blood vessels are surrounded by cuffs of inflammatory cells and contain marginating eosinophils, which migrated into their walls. (**C**) Mice challenged with OVA and treated with inhaled pitavastatin. (**D**) Mice challenged with OVA and treated with i.p. injections of dexamethasone. These panels depict the histological features representative of each condition in seven or eight mice per group. (**E**) Quantitative analysis of the degree of inflammatory cell infiltration in the lung sections, which was based on the methods developed by Myou and Lee^48, 49^. (**F**) Changes in the thickness of the reticular basement membrane of each group of mice with asthma. ***P < 0.001 compared with the control group; ^###^ *P* < 0.001 compared with the OVA group.

To assess the anti-inflammatory and anti-allergic effects of pitavastatin, we examined mouse lung specimens with a microscope. The results of the examination showed that minimal or no inflammation was present in the lungs of the mice in the control group (Fig. 4A). Conversely, the lungs of the mice in the OVA-challenged group displayed extensive inflammation, as well as severe peribronchial cuffing and eosinophil and lymphocyte infiltration. Red blood cells and mucus were visible in the alveolar cavities (Fig. 4B). However, the lungs of the mice in the pitavastatin- and dexamethasone-treated groups displayed significantly improved lung pathology compared with those of the mice in the OVA-challenged group. Specifically, the lungs in the groups in question displayed less extensive inflammatory cell infiltration and less red blood cells and secretions in their alveolar capillaries than the lungs in the OVA-challenged group. Moreover, pitavastatin and dexamethasone elicited similar improvements in the structural cells of the airways. The inflammation scores in the cells in the corresponding groups were lower than those of cells in the OVA-challenged group. Pitavastatin ameliorated OVA-induced increases in epithelial cell damage, as well as OVA-induced subepithelial fibrosis (Fig. 4C‒E).

**Figure 5:**
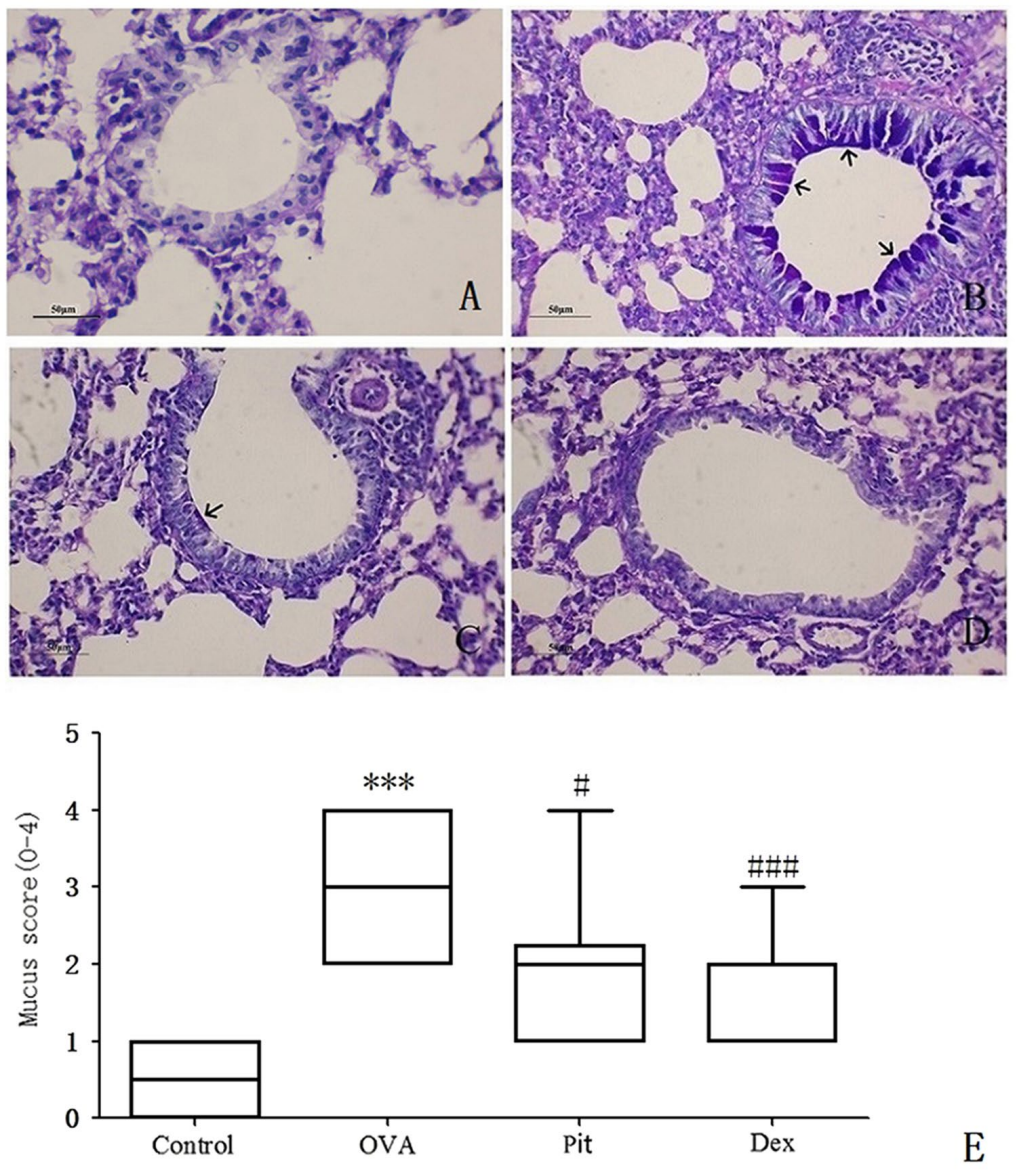
Histological images of airways stained with PAS for goblet cell (dark red in the lumen) (n = 10 per group) visualization. (**A**) Control mice treated with aerosolized PBS. (**B**) Mice challenged with OVA and treated with PBS. These mice displayed significantly increased numbers of goblet cells in their airway epithelia compared with control mice. (**C**) Mice challenged with OVA and treated with inhaled pitavastatin. (**D**) Mice challenged with OVA and treated with i.p. injections of dexamethasone. Mice treated with pitavastatin or dexamethasone displayed significantly less goblet cells than those challenged with OVA. These panels depict the histological features representative of each condition in seven or eight mice per group. (**E**) Kruskal-Wallis one-way analyses of mucus production in the lung sections were performed according to the methods developed by Myou and Lee^48, 49^. ***P < 0.001 compared with the control group; ^#^ *P* < 0.01 compared with the OVA group.

We stained the above lung sections with PAS to evaluate their levels of goblet cell hyperplasia. We observed noticeable differences in the sizes of the purple areas, i.e., the areas of lung tissue stained with PAS, among the four groups. We noted goblet cell hyperplasia and mucus overproduction in the bronchial passages of OVA-challenged mice. However, we noted a significantly lower number of goblet cells in the pitavastatin- and dexamethasone-treated groups than in the OVA-challenged group (Fig. 5A and B). As described in the Methods section, goblet cell hyperplasia scores were calculated for each experimental group. The mice in the OVA group had a staining score of 3.1, whereas the mice in the pitavastatin- and dexamethasone-treated groups had staining scores of 2.5 and 2.0, respectively (Fig. 5C–E).

### Pitavastatin normalizes IL-4, IL-17, and IFN-γ production in BALF in mice with asthma

**Figure 6:**
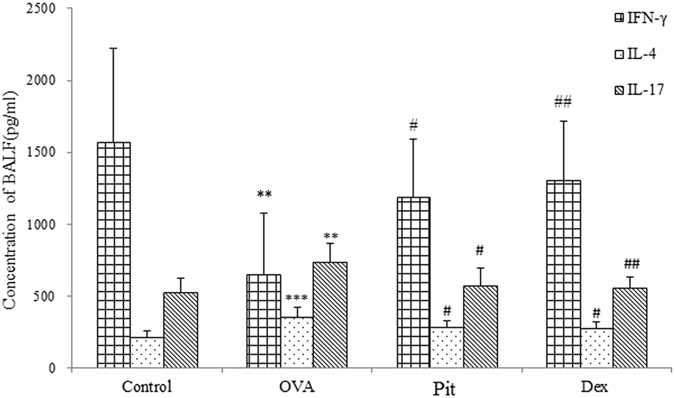
IL-4, IL-17, and IFN-γ levels in the BALF of mice treated with PBS, pitavastatin, or dexamethasone. Bronchoalveolar lavage samples were collected at 30 min after the last ovalbumin challenge. Control: Mice treated with aerosolized PBS. OVA: Mice challenged with OVA and treated with PBS. The numbers of goblet cells in the airway epithelia of these mice were significantly increased compared with those in the airway epithelial of control mice. Pit: Mice challenged with OVA and treated with inhaled pitavastatin. Dex: Mice challenged with OVA and treated with i.p. injections of dexamethasone. Values are expressed as the mean ± SD (n = 8‒10 per group). ***P < 0.001 compared with the control group; ^#^ *P* < 0.01 compared with the OVA group; ^##^ *P* < 0.05 compared with the OVA group.

To determine the anti-asthmatic effects of pitavastatin on cytokine levels in mice with asthma, we measured IL-4, IL-17, and IFN-γ production in BALF by ELISA, according to the manufacturer’s instructions. The mice in the OVA-challenged group exhibited significantly increased IL-4 and IL-17 levels and decreased IFN-γ levels in their BALF compared with the mice in the control group. However, IL-4 and IL-17 levels were decreased, and IFN-γ levels were increased in pitavastatin and dexamethasone-treated mice compared with OVA-challenged mice. Taken together, these findings indicate that the anti-inflammatory effects of pitavastatin and dexamethasone are probably mediated by the regulation of multiple inflammatory factors (Fig. 6).

### Effects of pitavastatin on IFN-γ and IL-4 protein expression in the lungs and mRNA expression in BALF

**Figure 7:**
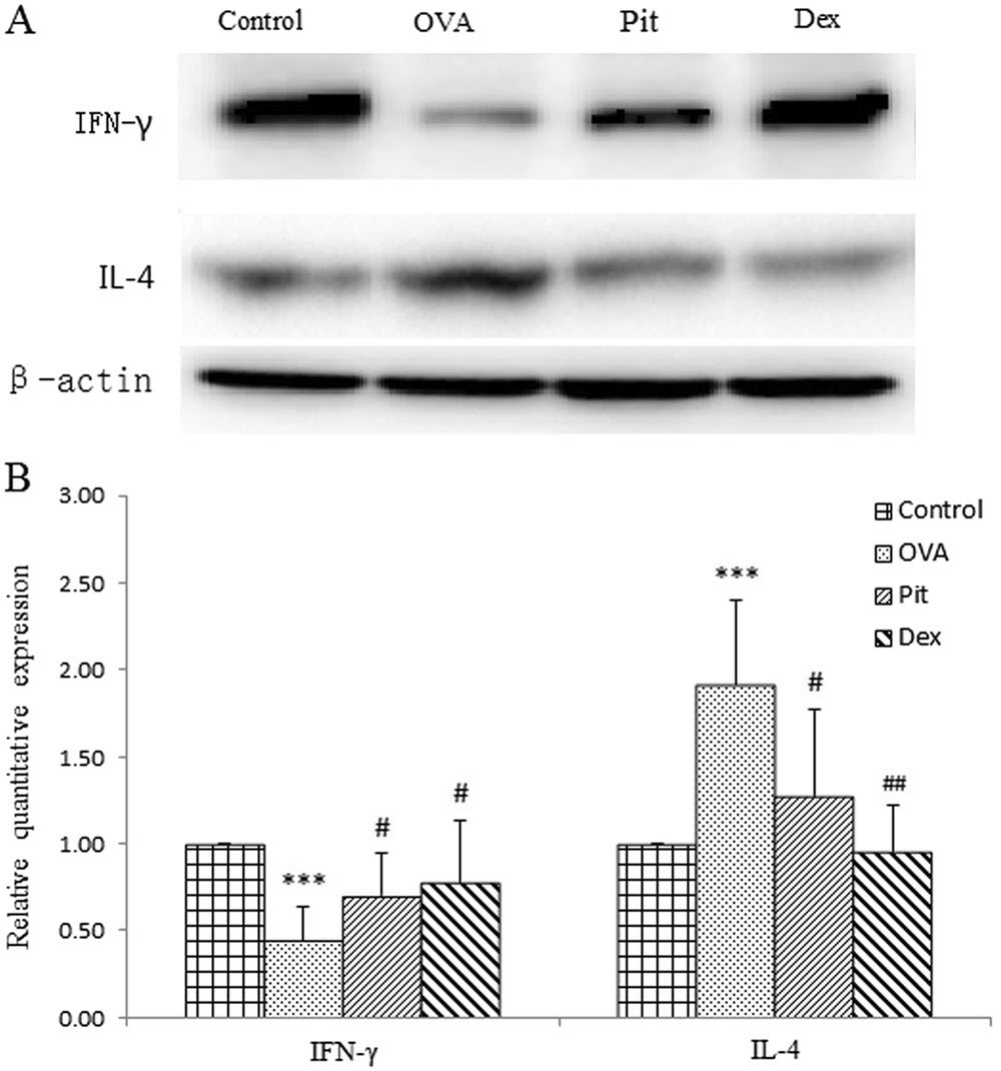
Effect of pitavastatin on IFN-γ and IL-4 expression in the lung tissues of asthmatic mice challenged with OVA. (**A**) Representative western blots showing IFN-γ expression in the different groups; (**B**) IFN-related optical densitometry results in the different groups. Control: Lung sections from mice treated with aerosolized PBS. OVA: Lung sections from mice challenged with OVA and treated with PBS. Pit and Dex: Lung sections from mice challenged with OVA and treated with pitavastatin and dexamethasone, respectively. ***P < 0.001 compared with the control group; ^#^ *P* < 0.05 compared with the OVA group; ^###^ *P* < 0.01 compared with the OVA group. The results from three independent experiments are similar. The data are expressed as the mean ± SD (n = 7). Full-length blots are presented in Supplementary Figure 1.

**Figure 8:**
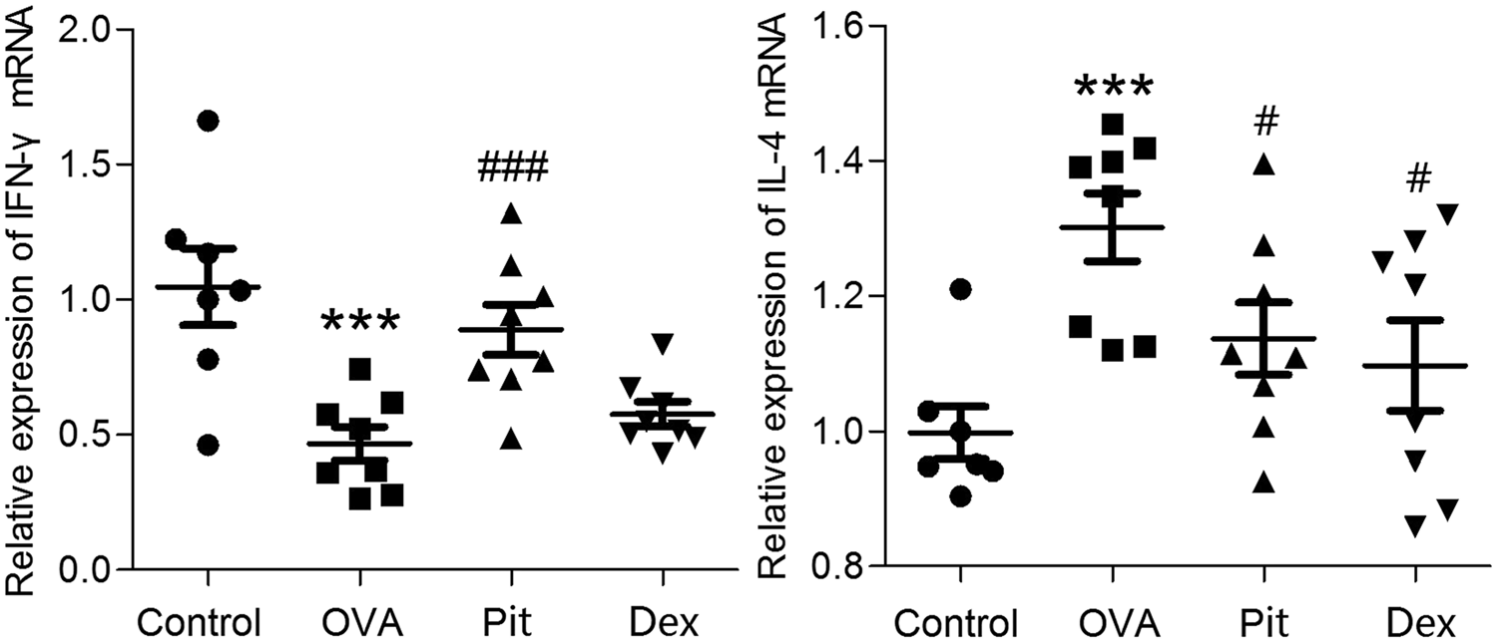
Effect of pitavastatin on IFN-γ and IL-4 mRNA expression, as determined by real time PCR. The relative mRNA expression levels of IFN-γ and IL-4 are expressed as the ratio of the mRNA level of the target gene to the mRNA levels of theβ-actin gene. The results represent the mean ± SD (*n* = 7–8). Control: Lung sections from mice treated with aerosolized PBS. OVA: Lung sections from mice challenged with OVA and treated with PBS. Pit and Dex: Lung sections from mice challenged with OVA and treated with pitavastatin and dexamethasone, respectively. ***P < 0.001 compared with the control group; ^#^ *P* < 0.05 compared with the OVA group; ^###^ *P* < 0.001 compared with the OVA group.

To evaluate the effects of pitavastatin as a treatment for allergic asthma on IFN-γ and IL-4 mRNA and protein expression, we assessed IFN-γ and IL-4 mRNA expression levels in BALF using RT-PCR and IFN-γ and IL-4 protein expression levels in the lungs through western blot analysis. OVA-challenged mice displayed significantly decreased IFN-γ mRNA expression levels in BALF and protein in the lungs compared with control mice. These decreases were reversed by pitavastatin, indicating that pitavastatin attenuates increases in IFN-γ levels in the lungs and BALF of OVA-challenged mice. And the mice in the dexamethasone-treated group displayed increased IFN-γ protein expression in the lungs compared with control mice (Figs 7 and 8). OVA-challenged mice displayed increased IL-4 protein expression in the lungs and mRNA expression levels in BALF compared with control mice. These increases can also been reversed by pitavastatin and dexamethasone (Figs 7 and 8).

## Discussion

The prevalence of asthma is rising. Thus, new agents that may be used to treat and prevent the disease are urgently needed, especially for patients who suffer from therapy-related side effects or respond poorly to conventional therapy^27, 28^. Several reports have shown that statins function as immune modulating agents and thus have protective effects on airway remodelling and airway inflammation in murine asthma models^14, 29, 30^. Statins have also been shown to have immunomodulatory effects in patients with asthma or chronic obstructive pulmonary disease (COPD)^31, 32^. Therefore, statins or similar agents may have beneficial effects in human patients with asthma. However, administering high doses of oral statins may have several adverse effects. Specifically, high doses of oral statins may cause myalgia, rhabdomyolysis, and increases in creatine phosphokinase levels^33^. Thus, statins that can be delivered as inhaled therapies must be developed. The results of this study indicate that pitavastatin has potential as an anti-asthma agent, as its pharmacological properties make it suitable for delivery as an inhaled agent.

Related to our findings, statins have been shown to selectively block pre-inflammation cytokines selectively, thereby decreasing mononuclear cell adhesion and impairing antigen presenting cell-mediated T cell activation^34^. In this study, pitavastatin significantly reduced AHR in mice with asthma. AHR is a critical evaluation tool with which asthma severity may be assessed (Fig. 1). Our histological results show that pitavastatin alleviates eosinophil and monocyte infiltration into the alveolar spaces of mice with asthma, decreases bronchial epithelium goblet cell numbers, and relieves pulmonary mucus secretion (Figs 4 and 5). Recruitment of eosinophils, lymphocytes and macrophages to the airways is a well-known characteristic of asthma, and the degree of eosinophil infiltration is correlated with the seriousness of patients’ conditions. These cells often play a major role in the induction of airway inflammation and hyper-responsiveness. The numbers of these cells in the BALF of pitavastatin-treated mice were decreased compared with those in the BALF of OVA-challenged mice (Fig. 2). These results show that inhaled pitavastatin may be used as a therapy for asthma prevention.

Imbalances in the relative levels of Thl and Th2 cytokines may contribute to the development of asthma^35, 36^. IL-4 and IFN-γ, which are secreted by Th2 and Th1 cells, respectively, have important immunological effects. IL-4, a Th2 cytokine, has been shown to promote the differentiation of Th0 cells into Th2 cells and IL-13-mediated IgE production by B cells, which enhances bronchiolar mucus production, as well as Th2 cytokine production. Moreover, IL-4 is also the principal determinant of eosinophil activation, recruitment and survival^37, 38^. IFN-γ promotes the differentiation of Th0 cells into Th1 cells, inhibits the cloning and differentiation of Th2 cells, and significantly improves the antigen presentation activity of macrophages. IFN-γ also inhibits IL-4 mRNA expression and reduces IL-4-induced IgE synthesis. A previous study showed that the percentage of IFN-γ-producing Th1 cells was significantly higher in patients with asthma than in control subjects^39, 40^. Thus, therapies that restore or maintain the balance between Thl and Th2 cells are considered important for the treatment of asthma. The results of the current study showed that pitavastatin exerted anti-inflammatory effects. Specifically, pitavastatin decreased IL-4 secretion and increased IFN-γ levels (Fig. 7).

Imbalances in the Treg/Th17 cell ratio have been identified in patients with asthma. We previously reported that the proportions of Treg and Th17 lymphocytes were altered in mice with induced asthma and that these alterations led to altered (lower) Treg/Th17 ratios and were thus indicative of the existence of an association between changes in lymphocyte levels and inflammation^41^. Our previous studies showed that pitavastatin inhibited IL-17 production in an asthma mouse model (Fig. 6). Therefore, we propose that pitavastatin may inhibit the secretion of Th17 cells, which secrete IL-17 to promote the inflammatory response and are associated with the pathogenesis of asthma.

FoxP3 is extremely important for CD4+ CD25+ T cell differentiation, proliferation potential, metabolism and function. FoxP3 expression levels have been confirmed to be low in patients with allergic asthma, resulting in impaired CD4+ CD25+ T cell differentiation and function^42, 43^. As immune modulating agents, statins have been shown to significantly influence the peripheral Treg pool in *vivo* and *vitro* ^44, 45^. Moreover, studies have reported that atorvastatin, a type of statin, can modulate the phenotypes of regulatory T cells in models of acute allergic asthma^46^. Additionally, it has been reported that increases in Treg cell levels likely contribute to the immunomodulatory effect of statins even in healthy individuals^47^. To test our hypothesis, we determined the numbers of CD4+ CD25+ Foxp3+ Treg cells in BALF in this study. Our results show that CD4+ CD25+ Foxp3+ T cell percentages were lower in the BALF of mice with asthma than in the BALF of control mice, illustrating that CD4+ CD25+ Foxp3+ Treg levels are diminished in the lungs of mice with asthma. However, CD4+ CD25+ Foxp3+ T cell percentages were significantly increased in the pitavastatin- and dexamethasone-treated groups compared with the OVA-challenged group (Fig. 3). Consistent with these findings, we found that CD4+ CD25+ Foxp3+ Treg depletion could induce asthma exacerbations. These findings are indicative of the importance of Treg cells in the protective effects of pitavastatin and suggest that the protective effects exerted by inhaled pitavastatin may be associated with CD4+ CD25+ Foxp3+ Treg cells in mouse models of asthma.

In conclusion, the findings of our study demonstrate that pitavastatin inhalation attenuates AHR, improves airway remodelling and lung pathology, increases CD4+ CD25+ Foxp3+ Treg cell numbers and balances the cytokines secreted by Th1 and Th2 cells in the lungs of mice with asthma and suggest that pitavastatin, whose properties make it suitable for delivery as an inhaled agent, may be a useful anti-inflammatory agent in the treatment of airway inflammatory diseases. Pitavastatin exerts its effects by regulating CD4+ CD25+ Foxp3+ Treg cell levels. Furthermore, we find that pitavastatin, on a wide variety of measures, similar to dexamethasone, greatly reduced or eliminated response to OVA challenge in a murine model of asthma. The differences in trend of effectiveness between these two treatments were minor. Since the clinical safety of pitavastatin inhalation has not been demonstrated, our results indicate that its effectiveness in a murine model of asthma is similar to that of glucocorticoids.

Agents targeting other asthma-related pathways and factors, such as Th17 and immunosuppressive cytokines (IL-10 and TGF-β), were not evaluated in this study. Moreover, as animal models have limitations with respect to the extent to which they approximate human diseases, the findings of this study must be validated by clinical studies in the future.

## Electronic supplementary material

Supplementary information
