# Supplementary material for: Anti-asthmatic effect of pitavastatin through aerosol inhalation is associated with CD4+ CD25+ Foxp3+ T cells in an asthma mouse model
Source: Sci Rep. 2017 Jul 20;7:6084. doi: 10.1038/s41598-017-06476-6 (PMC5519711; doi:10.1038/s41598-017-06476-6)
Supplement: Supplementary file 1 — Supplementary information [file 41598_2017_6476_MOESM1_ESM.pdf]

Article title:

Anti-asthmatic effect of pitavastatin through aerosol inhalation is associated with CD4+ CD25+ Foxp3+ T cells in an asthma mouse model

Song-quan Wu [#], Ru-hui Yang *[&], Guang-li Wang [$]

# Song-quan Wu, Master of Science in Immunology. Professor. Department of Immunology, College of medicine and health, Lishui University, Lishui, China.

* Ru-hui Yang, Ph. D. of Pharmacology. Associate professor. Department of Pharmacology, College of medicine and health, Lishui University, Lishui, China.

$ Guang-li Wang. Bachelor of Nursing. Experimenter. Department of Pharmacology, College of medicine and health, Lishui University, Lishui, China.

& Corresponding author: Ru-hui Yang, Ph. D. of Pharmacology. Associate professor. Department of Pharmacology, College of medicine and health, Lishui University, NO.1. Xueyuan Road, Lishui, Zhejiang Province, P.R. China, 323000.

Tel :(86)0578-2131183;

Fax: (86) 0571-28313178;

E-mail: yangruhui2008@163.com

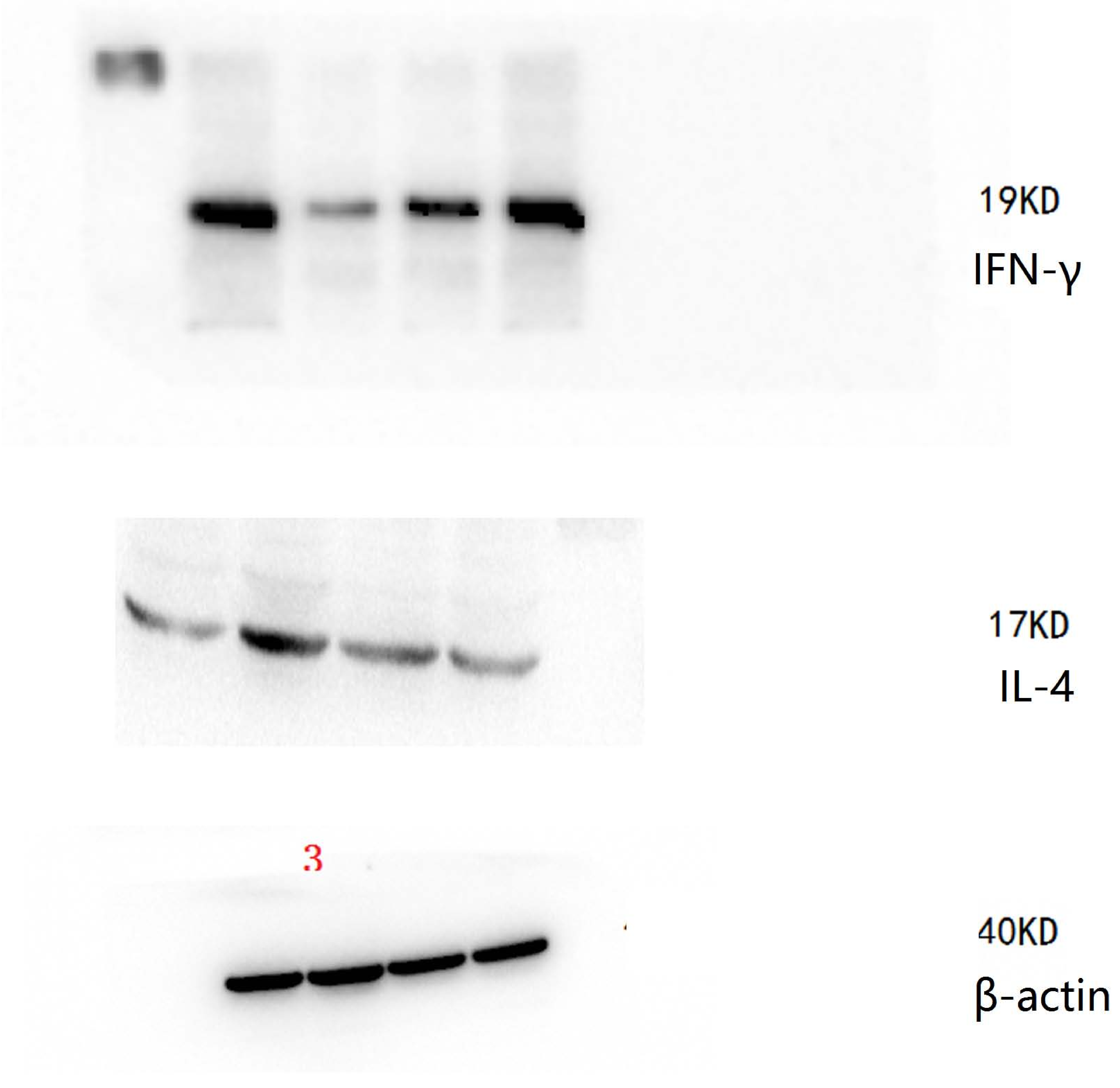
19KD
IFN-γ
17KD
IL-4
3
40KD
β-actin

2017/5/11 10:34

Report Information

User: BioRad/admin
File Name:
File Path:
Report Differs from Last Save: Yes

Gene Study File List

File Name	File Path	Date Created	Well Group Name	Run Type	Protocol Edited
admin_2017-04-29 12-16-59_BR001092-2.pcrd	F:\\\\\\\\-RT-qPCR170428\\20170429-IFN-	2017/4/29 14:01:12	All Wells	User-defined	No
admin_2017-04-29 14-16-40_BR001092-3.pcrd	F:\\\\\\\\-RT-qPCR170428\\20170429-IFN-	2017/4/29 16:01:23	All Wells	User-defined	No
admin_2017-04-29 10-25-07_BR001092-1.pcrd	F:\\\\\\\\-RT-qPCR170428\\20170429-IFN-	2017/4/29 12:10:36	All Wells	User-defined	No

Bar Chart

Analysis Mode: Normalized expression (Cq)
Chart Data: Relative to zero
Scaling options:
Chart Error: ± 1.0 SEMs

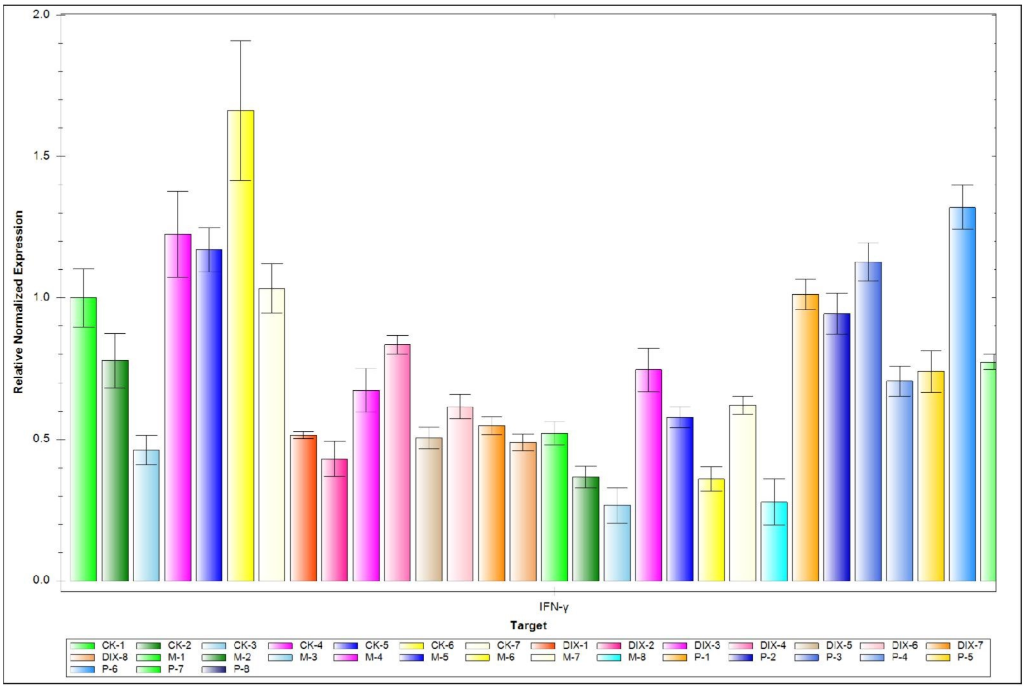

Target Names

Name	Full Name	Reference	Auto Efficiency	Efficiency
Actin	Actin	True	Yes	100.0%
IFN-	IFN-	False	Yes	100.0%

Sample Names

Name	Full Name	Control
CK-1	CK-1	Yes
CK-2	CK-2	No
CK-3	CK-3	No
CK-4	CK-4	No
CK-5	CK-5	No
CK-6	CK-6	No
CK-7	CK-7	No
DIX-1	DIX-1	No
DIX-2	DIX-2	No
DIX-3	DIX-3	No
DIX-4	DIX-4	No
DIX-5	DIX-5	No
DIX-6	DIX-6	No
DIX-7	DIX-7	No
DIX-8	DIX-8	No
M-1	M-1	No
M-2	M-2	No
M-3	M-3	No
M-4	M-4	No
M-5	M-5	No
M-6	M-6	No
M-7	M-7	No
M-8	M-8	No
P-1	P-1	No
P-2	P-2	No
P-3	P-3	No
P-4	P-4	No
P-5	P-5	No
P-6	P-6	No
P-7	P-7	No
P-8	P-8	No

Study Analysis - Bar Chart Data

Target	Sample	Ctrl	Expression	Expression SEM	Corrected Expression SEM	Mean Cq	Cq SEM
Actin	CK-1	*	N/A	N/A	N/A	21.72	0.06641
Actin	CK-2		N/A	N/A	N/A	21.71	0.02561
Actin	CK-3		N/A	N/A	N/A	21.02	0.06049
Actin	CK-4		N/A	N/A	N/A	21.94	0.06559
Actin	CK-5		N/A	N/A	N/A	23.33	0.02452
Actin	CK-6		N/A	N/A	N/A	24.02	0.03072

Study Analysis - Bar Chart Data

Target	Sample	Ctrl	Expression	Expression SEM	Corrected Expression SEM	Mean Cq	Cq SEM
Actin	CK-7		N/A	N/A	N/A	22.00	0.06296
Actin	DIX-1		N/A	N/A	N/A	19.45	0.02697
Actin	DIX-2		N/A	N/A	N/A	19.31	0.00539
Actin	DIX-3		N/A	N/A	N/A	19.78	0.00566
Actin	DIX-4		N/A	N/A	N/A	20.81	0.02584
Actin	DIX-5		N/A	N/A	N/A	20.35	0.08296
Actin	DIX-6		N/A	N/A	N/A	20.10	0.05691
Actin	DIX-7		N/A	N/A	N/A	19.18	0.03878
Actin	DIX-8		N/A	N/A	N/A	19.03	0.03700
Actin	M-1		N/A	N/A	N/A	20.80	0.07372
Actin	M-2		N/A	N/A	N/A	19.15	0.03124
Actin	M-3		N/A	N/A	N/A	20.87	0.09639
Actin	M-4		N/A	N/A	N/A	21.49	0.08209
Actin	M-5		N/A	N/A	N/A	18.86	0.07383
Actin	M-6		N/A	N/A	N/A	21.17	0.06432
Actin	M-7		N/A	N/A	N/A	19.50	0.06134
Actin	M-8		N/A	N/A	N/A	20.48	0.08270
Actin	P-1		N/A	N/A	N/A	21.26	0.04460
Actin	P-2		N/A	N/A	N/A	20.54	0.02591
Actin	P-3		N/A	N/A	N/A	21.03	0.02337
Actin	P-4		N/A	N/A	N/A	21.07	0.01591
Actin	P-5		N/A	N/A	N/A	19.68	0.02017
Actin	P-6		N/A	N/A	N/A	20.53	0.04567
Actin	P-7		N/A	N/A	N/A	20.40	0.01197
Actin	P-8		N/A	N/A	N/A	19.99	0.00797
IFN-	CK-1	*	1.00000	0.10330	0.10330	32.02	0.13342
IFN-	CK-2		0.77869	0.09707	0.09707	32.37	0.17801
IFN-	CK-3		0.46298	0.05238	0.05238	32.43	0.15161
IFN-	CK-4		1.22470	0.15170	0.15170	31.95	0.16623
IFN-	CK-5		1.17096	0.07803	0.07803	33.40	0.09296
IFN-	CK-6		1.66186	0.24690	0.24690	33.58	0.21212
IFN-	CK-7		1.03361	0.08739	0.08739	32.26	0.10447
IFN-	DIX-1		0.51527	0.01258	0.01258	30.71	0.02267
IFN-	DIX-2		0.43082	0.06251	0.06251	30.82	0.20923
IFN-	DIX-3		0.67391	0.07706	0.07706	30.65	0.16487
IFN-	DIX-4		0.83412	0.03248	0.03248	31.37	0.04988
IFN-	DIX-5		0.50469	0.03811	0.03811	31.64	0.07059
IFN-	DIX-6		0.61560	0.04264	0.04264	31.09	0.08214
IFN-	DIX-7		0.54863	0.03089	0.03089	30.34	0.07138
IFN-	DIX-8		0.48930	0.02987	0.02987	30.36	0.07992
IFN-	M-1		0.52207	0.04128	0.04128	32.04	0.08706
IFN-	M-2		0.36801	0.03800	0.03800	30.89	0.14567
IFN-	M-3		0.26632	0.06326	0.06326	33.08	0.32883
IFN-	M-4		0.74622	0.07726	0.07726	32.21	0.12480
IFN-	M-5		0.57755	0.03668	0.03668	29.95	0.05426
IFN-	M-6		0.36027	0.04246	0.04246	32.94	0.15742
IFN-	M-7		0.62113	0.03235	0.03235	30.49	0.04339
IFN-	M-8		0.27871	0.08189	0.08189	32.62	0.41575
IFN-	P-1		1.01310	0.05411	0.05411	31.54	0.06283

Study Analysis - Bar Chart Data

Target	Sample	Ctrl	Expression	Expression SEM	Corrected Expression SEM	Mean Cq	Cq SEM
IFN-	P-2		0.94467	0.07149	0.07149	30.92	0.10606
IFN-	P-3		1.12714	0.06665	0.06665	31.15	0.08205
IFN-	P-4		0.70623	0.05365	0.05365	31.87	0.10844
IFN-	P-5		0.74003	0.07262	0.07262	30.41	0.14013
IFN-	P-6		1.32005	0.07828	0.07828	30.43	0.07234
IFN-	P-7		0.77376	0.02711	0.02711	31.06	0.04911
IFN-	P-8		0.48689	0.04674	0.04674	31.33	0.13816

Inter-run Calibration

Actin

IFN-

2017/5/11 10:09

Report Information

User: BioRad/admin
File Name:
File Path:
Report Differs from Last Save: Yes

Gene Study File List

File Name	File Path	Date Created	Well Group Name	Run Type	Protocol Edited
test 2.pcrd	F:\\\\\\\\-RT-qPCR170428\\IL-4\1. - 2	2017/4/28 15:33:49	All Wells	User-defined	No
test 4.pcrd	F:\\\\\\\\-RT-qPCR170428\\IL-4\1. - 2	2017/4/28 17:44:39	All Wells	User-defined	No
test 5.pcrd	F:\\\\\\\\-RT-qPCR170428\\IL-4\1. - 2	2017/4/28 17:43:55	All Wells	User-defined	No
test 3.pcrd	F:\\\\\\\\-RT-qPCR170428\\IL-4\1. - 2	2017/4/28 16:13:14	All Wells	User-defined	No
test 1.pcrd	F:\\\\\\\\-RT-qPCR170428\\IL-4\1. - 2	2017/4/28 14:39:13	All Wells	User-defined	No

Bar Chart

Analysis Mode: Normalized expression (Cq)
Chart Data: Relative to zero
Scaling options:
Chart Error: ± 1.0 SEMs

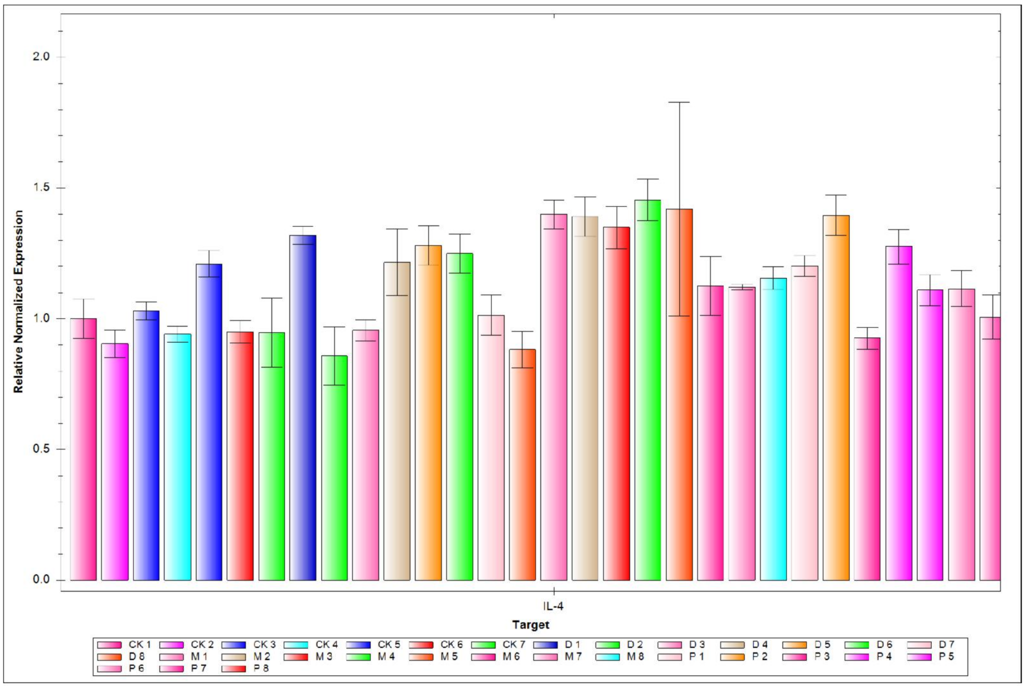

Target Names

Name	Full Name	Reference	Auto Efficiency	Efficiency
Actin	Actin	True	Yes	100.0%
IL-4	IL-4	False	Yes	100.0%

Sample Names

Name	Full Name	Control
CK 1	CK 1	Yes
CK 2	CK 2	No
CK 3	CK 3	No
CK 4	CK 4	No
CK 5	CK 5	No
CK 6	CK 6	No
CK 7	CK 7	No
D 1	D 1	No
D 2	D 2	No
D 3	D 3	No
D 4	D 4	No
D 5	D 5	No
D 6	D 6	No
D 7	D 7	No
D 8	D 8	No
M 1	M 1	No
M 2	M 2	No
M 3	M 3	No
M 4	M 4	No
M 5	M 5	No
M 6	M 6	No
M 7	M 7	No
M 8	M 8	No

Sample Names

Name	Full Name	Control
P 1	P 1	No
P 2	P 2	No
P 3	P 3	No
P 4	P 4	No
P 5	P 5	No
P 6	P 6	No
P 7	P 7	No
P 8	P 8	No

Study Analysis - Bar Chart Data

Target	Sample	Ctrl	Expression	Expression SEM	Corrected Expression SEM	Mean Cq	Cq SEM
Actin	CK 1	*	N/A	N/A	N/A	21.68	0.08860
Actin	CK 2		N/A	N/A	N/A	22.70	0.02179
Actin	CK 3		N/A	N/A	N/A	22.05	0.02570
Actin	CK 4		N/A	N/A	N/A	22.03	0.01718
Actin	CK 5		N/A	N/A	N/A	22.01	0.05656
Actin	CK 6		N/A	N/A	N/A	22.38	0.05065
Actin	CK 7		N/A	N/A	N/A	22.76	0.14345
Actin	D 1		N/A	N/A	N/A	21.77	0.02644
Actin	D 2		N/A	N/A	N/A	21.51	0.16734
Actin	D 3		N/A	N/A	N/A	23.97	0.03954
Actin	D 4		N/A	N/A	N/A	22.73	0.15025
Actin	D 5		N/A	N/A	N/A	21.83	0.08094
Actin	D 6		N/A	N/A	N/A	24.11	0.04174
Actin	D 7		N/A	N/A	N/A	22.36	0.08200
Actin	D 8		N/A	N/A	N/A	21.96	0.09894
Actin	M 1		N/A	N/A	N/A	22.96	0.05203
Actin	M 2		N/A	N/A	N/A	23.21	0.03222
Actin	M 3		N/A	N/A	N/A	23.29	0.00981
Actin	M 4		N/A	N/A	N/A	21.98	0.07381
Actin	M 5		N/A	N/A	N/A	22.69	0.04854
Actin	M 6		N/A	N/A	N/A	21.91	0.10585
Actin	M 7		N/A	N/A	N/A	22.32	0.01236
Actin	M 8		N/A	N/A	N/A	20.47	0.01005
Actin	P 1		N/A	N/A	N/A	21.87	0.01413
Actin	P 2		N/A	N/A	N/A	23.11	0.01317
Actin	P 3		N/A	N/A	N/A	21.57	0.04791
Actin	P 4		N/A	N/A	N/A	23.10	0.05821
Actin	P 5		N/A	N/A	N/A	22.16	0.04557
Actin	P 6		N/A	N/A	N/A	21.83	0.04612
Actin	P 7		N/A	N/A	N/A	23.16	0.03606
Actin	P 8		N/A	N/A	N/A	23.29	0.93618
IL-4	CK 1	*	1.00000	0.07572	0.07572	25.77	0.06390
IL-4	CK 2		0.90386	0.05300	0.05300	26.93	0.08174
IL-4	CK 3		1.02983	0.03479	0.03479	26.09	0.04141
IL-4	CK 4		0.94083	0.03017	0.03017	26.20	0.04296

Study Analysis - Bar Chart Data

Target	Sample	Ctrl	Expression	Expression SEM	Corrected Expression SEM	Mean Cq	Cq SEM
IL-4	CK 5		1.21060	0.05099	0.05099	25.82	0.02222
IL-4	CK 6		0.95094	0.04310	0.04310	26.54	0.04136
IL-4	CK 7		0.94778	0.13289	0.13289	26.92	0.14261
IL-4	D 1		1.32019	0.03518	0.03518	25.46	0.02791
IL-4	D 2		0.85849	0.11139	0.11139	25.82	0.08389
IL-4	D 3		0.95589	0.03944	0.03944	28.12	0.04448
IL-4	D 4		1.21608	0.12810	0.12810	26.53	0.02279
IL-4	D 5		1.28129	0.07539	0.07539	25.56	0.02558
IL-4	D 6		1.24964	0.07481	0.07481	27.88	0.07561
IL-4	D 7		1.01358	0.07640	0.07640	26.43	0.07143
IL-4	D 8		0.88274	0.06991	0.06991	26.23	0.05716
IL-4	M 1		1.39909	0.05467	0.05467	26.57	0.02169
IL-4	M 2		1.39086	0.07550	0.07550	26.82	0.07138
IL-4	M 3		1.34907	0.08113	0.08113	26.94	0.08620
IL-4	M 4		1.45479	0.07938	0.07938	25.53	0.02738
IL-4	M 5		1.41929	0.40887	0.40887	26.27	0.41277
IL-4	M 6		1.12555	0.11227	0.11227	25.82	0.09750
IL-4	M 7		1.12113	0.01130	0.01130	26.24	0.00767
IL-4	M 8		1.15547	0.04257	0.04257	24.35	0.05219
IL-4	P 1		1.20191	0.04009	0.04009	25.69	0.04600
IL-4	P 2		1.39639	0.07601	0.07601	26.72	0.07742
IL-4	P 3		0.92559	0.04232	0.04232	25.77	0.04535
IL-4	P 4		1.27599	0.06532	0.06532	26.83	0.04544
IL-4	P 5		1.10914	0.06019	0.06019	26.10	0.06366
IL-4	P 6		1.11470	0.06822	0.06822	25.76	0.07530
IL-4	P 7		1.00670	0.08468	0.08468	27.24	0.11587
IL-4	P 8		1.06710	0.99669	0.99669	27.28	0.96920

Inter-run Calibration

Actin

IL-4
